# Enhancing outreach for persons with serious mental illness: 12-month results from a cluster randomized trial of an adaptive implementation strategy

**DOI:** 10.1186/s13012-014-0163-3

**Published:** 2014-12-28

**Authors:** Amy M Kilbourne, Daniel Almirall, David E Goodrich, Zongshan Lai, Kristen M Abraham, Kristina M Nord, Nicholas W Bowersox

**Affiliations:** VA Center for Clinical Management Research, VA Ann Arbor Healthcare System, 2215 Fuller Road, Mailstop 152, Ann Arbor, MI 48105 USA; Department of Psychiatry, University of Michigan Medical School, North Campus Research Complex, 2800 Plymouth Road, Building 16, Ann Arbor, MI 48109-2800 USA; Institute for Social Research, University of Michigan, 426 Thompson Street, Ann Arbor, MI 48104-2321 USA; University of Detroit Mercy, 4001 West McNichols Road, Detroit, MI 48221-3038 USA

**Keywords:** Mental disorders, Implementation science, Population health

## Abstract

**Background:**

Few implementation strategies have been empirically tested for their effectiveness in improving uptake of evidence-based treatments or programs. This study compared the effectiveness of an immediate versus delayed enhanced implementation strategy (Enhanced Replicating Effective Programs (REP)) for providers at Veterans Health Administration (VA) outpatient facilities (sites) on improved uptake of an outreach program (Re-Engage) among sites not initially responding to a standard implementation strategy.

**Methods:**

One mental health provider from each U.S. VA site (*N* = 158) was initially given a REP-based package and training program in Re-Engage. The Re-Engage program involved giving each site provider a list of patients with serious mental illness who had not been seen at their facility for at least a year, requesting that providers contact these patients, assessing patient clinical status, and where appropriate, facilitating appointments to VA health services. At month 6, sites considered non-responsive (*N* = 89, total of 3,075 patients), defined as providers updating documentation for less than <80% of patients on their list, were randomized to two adaptive implementation interventions: Enhanced REP (provider coaching; *N* = 40 sites) for 6 months followed by Standard REP for 6 months; versus continued Standard REP (*N* = 49 sites) for 6 months followed by 6 months of Enhanced REP for sites still not responding. Outcomes included patient-level Re-Engage implementation and utilization.

**Results:**

Patients from sites that were randomized to receive Enhanced REP immediately compared to Standard REP were more likely to have a completed contact (adjusted OR = 2.13; 95% CI: 1.09–4.19, *P* = 0.02). There were no differences in patient-level utilization between Enhanced and Standard REP sites.

**Conclusions:**

Enhanced REP was associated with greater Re-Engage program uptake (completed contacts) among sites not responding to a standard implementation strategy. Further research is needed to determine whether national implementation of Facilitation results in tangible changes in patient-level outcomes.

**Trial registration:**

ISRCTN: ISRCTN21059161

**Electronic supplementary material:**

The online version of this article (doi:10.1186/s13012-014-0163-3) contains supplementary material, which is available to authorized users.

## Background

There are substantial delays between the time that treatments are proven effective and when they are routinely implemented in practice. This research-to-practice gap is especially pertinent to the delivery of treatments for persons with serious mental illness (e.g., schizophrenia or related disorders, bipolar disorder), which are associated with substantial functional impairment, medical burden [1-3], health costs [4,5], and premature mortality [6-11].

There has been growing interest in developing and testing implementation strategies that more rapidly and effectively translate programs into routine care settings [12]. Implementation strategies are operationalized techniques based on an underlying framework or theory that are designed to enhance the uptake of effective programs across different health-care settings [13]. A variety of implementation strategies and supporting frameworks exist [14-18], with relative advantage conferred on those that are (a) theory-based, (b) described in highly specified operational terms, and (c) applicable across different care settings.

Replicating Effective Programs (REP) is a previously operationalized implementation strategy based on the Centers for Disease Control and Prevention’s Research-to-Practice Framework [16,17,19]. Derived from Social Learning Theory [20] and Rogers’ diffusion model [21], REP consists of three central operational components: program “packaging” (i.e., translation and dissemination of treatment materials into user-friendly language), structured training for providers, and brief technical assistance for providers focused on the technical aspects of program implementation. These three components in combination compared to package dissemination alone resulted in improved uptake of HIV prevention intervention programs in AIDS service organizations [19,22].

Although REP employs key tactical strategies that can promote treatment adoption [19,23], it is likely inadequate for more complex programs that involve more than a single behavioral intervention or provider. Notably, care for persons with serious mental illness is often managed across different provider and organizational boundaries that can make implementation challenging [24]. Moreover, competing demands on providers and the need to garner support from multiple levels of providers and leaders require implementation strategies beyond training, such as coaching in strategic thinking to enable leadership support and adaptation across sites [25-27].

Hence, REP was recently enhanced to address provider and system-level barriers to adoption [16] by including additional strategies that engage both service leaders and frontline providers [28,29]. Enhanced REP includes ongoing Facilitation based on the Promoting Action on Research Implementation in Health Services (PARiHS) framework [23,30-32]. Facilitation is a systematic and iterative process in which technical experts promote program uptake by building relationships with providers and working with them to identify and mitigate barriers to program adoption, often using one-on-one consultation by phone [28,33]. Facilitation was chosen as additional implementation strategy to REP because of its relative feasibility in application across a national network of sites, as well as similarity in theoretical underpinning to other implementation strategies that focus on improving the uptake of psychosocial interventions [34,35].

In a previous study, Enhanced REP with added Facilitation compared to Standard REP was associated with improved fidelity to an effective mental health collaborative care program in community-based practices [36]. However, this study did not address key questions that would inform a larger rollout of Enhanced REP, notably, whether adding Facilitation to sites that are initially non-responsive to Standard REP and whether more immediate versus delayed implementation of Facilitation lead to improved program uptake.

This current study reports 12-month findings from a national cluster randomized intervention study comparing two adaptive implementation strategies among sites that are initially non-responsive to Standard REP: one providing Enhanced REP immediately versus another delaying the provision of Enhanced REP only for sites that remain non-responsive 6 months later. The primary outcome was uptake of an outreach program for patients with serious mental illness (SMI). The program, Re-Engage, involved mental health providers identifying Veterans with SMI who had dropped out of care (i.e., not seen by a provider for at least 1 year), documenting their current status, and providing outreach to assess clinical need and to schedule health-care appointments if needed. Developed by the Veterans Health Administration (VA), Re-Engage was associated with improved access to care and reduced mortality in a previous effectiveness trial [37]. Re-Engage became a nationally mandated program in the VA in January 2012 [38] and hence, provided an ideal opportunity in which to compare the effectiveness of Enhanced and Standard REP on its uptake and patient outcomes.

The study’s primary hypothesis is that among patients from non-responsive sites, those from sites randomized to the adaptive intervention Enhanced REP for 6 months followed by Standard REP would be more likely to receive Re-Engage program components within 12 months compared to those from non-responsive sites randomized to continue Standard REP for 6 months and then receiving Enhanced REP after 6 months (for sites that remain non-responsive). Implementation of Re-Engage program components was defined as updated documentation of patients’ current status in a web-based registry, attempts to contact the patient (i.e., attempted contact), and successfully contacting the patient (i.e., completed contact). Secondary aims of the study included assessing differences in patient utilization occurred between the adaptive interventions.

## Methods

The rationale and protocol for this two-arm cluster randomized controlled implementation trial has been described elsewhere [39]. This study was reviewed and approved by the local Institutional Review Board and was registered as a clinical trial (Current Controlled Trials ISRCTN21059161).

### Setting

The study included all U.S. VA facilities (sites) that were required by the VA National Policy Directive [38] to assign a designated VA mental health provider to implement Re-Engage, with the adaptive implementation trial focusing on sites that did not initially implement Re-Engage in response to receiving 6 months Standard REP alone.

### Re-Engage treatment program

Described elsewhere [37,40], Re-Engage is a national VA outreach program that consists of risk assessment (i.e., identifying the patients’ current status including clinical care needs and current disposition) and outreach services (i.e., attempting to contact patients and invite them back to receive health services). Re-Engage was developed by VA Office of Medical Inspector to assist patients with a diagnosis of serious mental illness (SMI; schizophrenia or related disorders, or bipolar disorder) who had dropped out of care to re-connect to health services if needed. In a previous national quality improvement study, 72% of Veterans with SMI who had not been seen in care for at least 1 year returned for VA care. The mortality rate of returning patients was significantly lower than that for patients not returning to care (0.5% versus 3.9%; adjusted odds ratio = 5.8; *P* < .001), after demographic and clinical factors were controlled for in the analyses [37,40]. As a result, VA leadership desired to have the Re-Engage program rolled out via a National Directive as part of standard care, and the goal of testing implementation strategies (i.e., Facilitation) was to ensure that the program was implemented as intended in this national rollout [38].

### Re-Engage target population and site eligibility

For this study, the unit of the implementation strategy intervention was the site. Initially, the responsibility for implementing Re-Engage was the designated mental health provider at each eligible VA site (Local Recovery Coordinator) [41]. Local Recovery Coordinators are social workers, psychologists, nurses, physicians, or marriage and family therapists, but at the time of this study, the vast majority (96%) of them were licensed social workers or psychologists, and less than 4% were other health professionals (two were Physician Assistants and three were Physicians).

A VA facility (site) was eligible for the current trial if it was included in the national VA Re-Engage program. VA sites were included in the national Re-Engage program if they were 1) within the 50 United States or Puerto Rico, 2) were required, per VA policy [41], to have a Local Recovery Coordinator to implement the national Re-Engage Directive, and 3) had at least one VA patient diagnosed with an ICD-9 diagnosis of schizophrenia or related disorder or bipolar disorder who was lost to care. Lost to care was defined as whether the patient had been seen at the facility between fiscal year (FY) 2008–2009 but had no subsequent outpatient visits or an inpatient stay of less than 2 days as of January 2012. There were a total of 158 sites eligible for Re-Engage, of which 139 were medical centers (i.e., with hospital beds) and 19 were community-based outpatient clinics.

### Cluster randomized implementation intervention trial procedures

Re-Engage was implemented based on the following steps. First, all eligible sites received Standard REP for 6 months to support the implementation of Re-Engage starting March 1, 2012, when VA disseminated the National Directive [38]. Standard REP included disseminating the list of veterans identified as having dropped out of care along with a package that included a web-based clinical registry in order to document patients’ current status and their attempts to contact the patient, and instructions for providing Re-Engage outreach services. The Local Recovery Coordinator at each eligible VA site received a list of names and last known contact information of eligible veterans from the VA national mental health program office who had dropped out of care and were last seen at their site. Veterans who were eligible for Re-Engage [37,42] and who were on the providers’ list 1 had at least one diagnosis of schizophrenia or related disorder (International Classification of Diseases, Ninth Revision, Clinical Modification (ICD-9-CM) codes 295.0–295.4; 295.6–295.9), or bipolar disorder (ICD-9-CM codes 296.0–296.8) recorded in an inpatient or outpatient visit in FY 2008 or FY 2009, 2) had not been seen in VA care for at least 1 year (i.e., dropped out of care: defined as no recorded outpatient visits in the past year), 3) had at least one inpatient visit prior to drop out, 4) were less than 65 years of age (i.e., less likely to be in a nursing home or covered by Medicare services), and 5) were still alive as of March 2012 based on currently available mortality information from the VA Beneficiary Identification and Records Locater Subsystem, the Social Security Administration Death Master File, and the National Death Index [43].

In addition to the list- and web-based registry, Local Recovery Coordinators at each site also received a guide describing the Re-Engage program and were provided training via conference calls and offered brief technical assistance for 6 months [39]. During this period, Local Recovery Coordinators were asked to identify and document their patients’ current disposition based on the pre-generated list of those who had dropped out of care. Specifically, providers attempted to contact patients from the outreach list and if successfully contacted, assess their clinical need and schedule a VA appointment if the Veteran desired health care.

**Figure 1:**
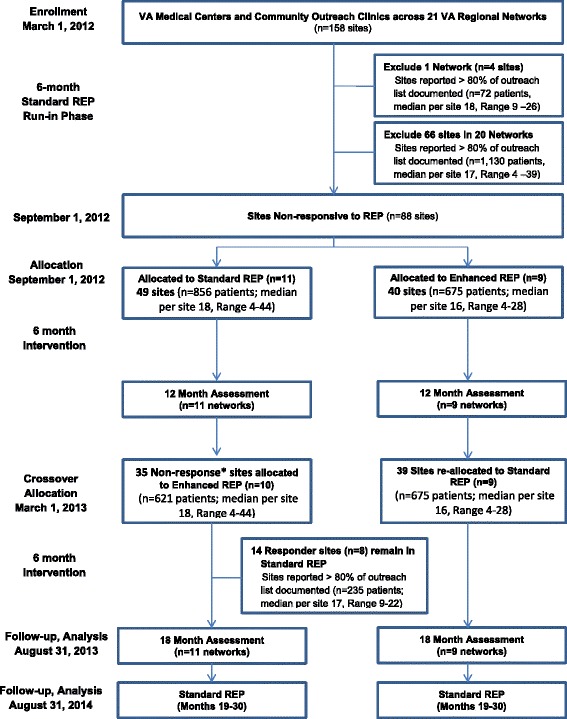
**CONSORT diagram for cluster randomized trial of an adaptive implementation strategy.**

Sites with inadequate implementation of Re-Engage (i.e., non-responding sites) as of September 1, 2012 were then identified based on a previously established eligibility criterion for inadequate implementation of Re-Engage and randomized to receive Enhanced REP or continue Standard REP from September 1, 2012 through February 28, 2013 (see Figure 1). Inadequate implementation of Re-Engage was defined as having <80% of patients who were previously identified as lost to care and were assigned to the given facility with an updated documentation of current clinical status in the web-based registry. This previously established measure is considered a core component of the Re-Engage program because it is an indicator of whether the Local Recovery Coordinator at each site reviewed the list to assess clinical status and attempted to locate the patient. This measure is also most likely impacted by individual providers and could be directly addressed through implementation interventions. A cut-point of 80% was selected because it is a standard definition used to determine adequate adherence to practice guidelines [44]. No differences in site characteristics were observed among the responding and non-responding sites (Table 1). In addition, a total of 3,075 patients were identified from the 89 sites (mean number of patients per site = 36; SD = 25; range 4 to 145 patients). Patients from non-responding sites were slightly more likely to be older and have a diagnosis of schizophrenia or related disorder (Table 1).

**Table 1:** **Characteristics of study sites and patients**

	**All Sites (** ***N*** **= 158)**	**Initially responsive sites (** ***N*** **= 69)** ^**a**^	**Initially non-responsive sites (** ***N*** **= 89)**		
			**Overall (** ***N*** **= 89 sites)**	**Randomized to Enhanced REP (** ***N*** **= 40 sites)**	**Randomized to Standard REP (** ***N*** **= 49 sites)**
Site characteristics					
Region	*N* (%)	*N* (%)	*N* (%)	*N* (%)	*N* (%)
Northeast	41 (25.95)	19 (27.54)	22 (24.72)	12 (30.00)	10 (20.41)
Midwest	38 (24.05)	15 (21.74)	23 (25.84)	13 (32.50)	10 (20.41)
South	45 (28.48)	23 (33.33)	22 (24.72)	8 (20.00)	14 (28.57)
West	34 (21.52)	12 (17.39)	22 (24.72)	7 (17.50)	15 (30.61)
Site provides outpatient care only	19 (12.03)	9 (13.04)	10 (11.24)	4 (10.00)	6 (12.24)
Mean total number of patients at site as of fiscal year 2012	40,858 (21,346)	41,943 (22,867)	40,016 (20,179)	41,427 (18,216)	38,865 (21,770)
Mean number of patients diagnosed with serious mental illness lost to care on site list	17 (6)	17 (6)	17 (7)	17 (6)	17 (7)
**Patient characteristics**	***N*** **= 5,047 patients**	***N*** **= 1,972 patients**	***N*** **= 3,075 patients**	***N*** **= 1,543 patients**	***N*** **= 1,532 patients**
	***N*** **(%)**	***N*** **(%)**	***N*** **(%)**	***N*** **(%)**	***N*** **(%)**
Male	4,550 (90.2)	1,768 (89.7)	2,782 (90.5)	1,405 (91.1)	1,377 (89.9)
Black	886 (17.6)	322 (16.3)	564 (18.3)	329 (21.3)	235 (15.3)*
Married	1,363 (27.0)	548 (27.8)	815 (26.5)	405 (26.3)	410 (26.8)
Service Connected	1,422 (28.2)	561 (28.5)	861 (28.0)	424 (27.5)	437 (28.5)
Homeless	650 (12.9)	227 (11.5)	423 (13.8)	229 (14.8)	194 (12.7)
Schizophrenia Diagnosis	2,017 (39.9)	739 (37.5)	1,278 (41.6)*	683 (44.3)	595 (38.8)*
Last encounter at Site was inpatient	247 (4.9)	109 (5.5)	138 (4.5)	69 (4.5)	69 (4.5)
	**Mean (SD)**	**Mean (SD)**	**Mean (SD)**	**Mean (SD)**	**Mean (SD)**
Age, mean (SD)	55.1 (14.3)	54.4 (14.3)	55.5 (14.4)*	55.5 (14.1)	55.6 (14.6)
Number of comorbidities, mean (SD)	1.4 (1.7)	1.5 (1.7)	1.4 (1.6)	1.4 (1.6)	1.5 (1.7)

^**a**^Non-responsive sites were defined as <80% of patients at the site with an updated documentation of current status; Responsive sites had ≥80% documentation of current status, as of March 2012.

*P value < 0.05 based on Rao-Scott chi-square test for categorical variables and two-tailed *t* test for continuous variables.

### Randomization procedures

Sites having successfully implemented Re-Engage by August 31, 2012 continued to receive Standard REP (these sites are not part of the present study). Sites that had not adequately implemented Re-Engage (i.e., non-responding sites) were included in the present study and were randomized at the regional level by integrated service networks. In the VA health-care system, sites reside within 21 regional VA integrated service networks (VISNs) representing the 50 United States and territories. A total of 89 sites (56.3%) from 20 of the 21 VISNs were non-responders to Standard REP support to implement Re-Engage (<80% of patients with updated documentation of current status). The VISNs of these sites were stratified by geographic region and randomized with equal probability to two implementation interventions (Enhanced REP or continued Standard REP described below). Among the 20 regional networks (VISNs), 9 VISNs that included 40 sites were randomized to begin with Enhanced REP and 11 VISNs that included 49 sites were randomized to continue with Standard REP.

We chose to randomize at the regional network level (VISN) and not the site level because VISNs are typically authorized to set policies including allocation of clinical resources across VA facilities based on their own budget. Moreover, each regional network has a designated mental health leader who is responsible for monitoring mental health program activities among the network’s individual sites and who would likely be contacted as part of the Facilitation implementation strategy (Table 2). Hence, randomization was conducted at the regional network level in the event that individual providers interacted with VISN-level leadership to garner support for the implementation of Re-Engage at their site, thereby minimizing potential for contamination across sites.

**Table 2:** **Components and timeline of the two adaptive interventions: Enhanced and Standard Replicating Effective Programs (REP)**

**Time**	**All Sites—Standard REP**	
March 1, 2012	**Re-Engage package**: Implementation guide disseminated to all providers at eligible sites, describing the Re-Engage program, a list of frequently asked questions, sample documents for program tasks, program policies, data security, and related research.	
	**Re-Engage training**: Three 1.5-h national conference call trainings of mental health providers on how to conduct program. Program materials made available on mental health provider website.	
	**Technical assistance**: Ongoing assistance via 1-h biweekly conference calls led by study staff for mental health providers to answer technical questions on Re-Engage program implementation, and study staff were available on an *ad hoc* basis to answer questions from individual providers. Monthly reports were generated to track Re-Engage uptake (% patients with updated current status documented).	
September 1, 2012	**Non-responsive sites (<80% patients with updated documentation of current status) randomized to Enhanced or Standard REP**	
September 1, 2012–March 1, 2013	**Enhanced REP**	**Standard REP**
	**Needs assessment**: Facilitators gather information from various sources (monthly evaluation reports, regional mental health leadership, mental health providers, VA national mental health leadership) to identify potential facility-specific barriers and facilitators to implementation.	**As Described Above**
	**Ongoing support**: Weekly phone calls with Facilitators, Technical Assistance staff, and VA national mental health leaders. Facilitators maintain open communication with VA leaders regarding implementation nationally and at specific sites through these phone calls and email communication. Facilitators also maintain ongoing contact with one another through separate weekly meetings.	
	**Garner local and regional support**: Facilitators initiate contact with regional mental health leadership affiliated with local sites, providing information regarding Re-Engage program implementation and added value. Maintain ongoing contact and request support from regional leadership as indicated.	
	**Identify barriers/facilitators to Re-Engage implementation**: Facilitators and mental health providers hold monthly calls for 6 months and collaboratively identify each facility’s specific challenges (e.g., time, resources) to program implementation as well as potential assets (e.g., consistency with other initiatives, support from regional leadership).	
	**Collectively develop action plans**: Facilitators assist mental health providers in identifying what specific actions they can take to implement program.	
	**Feedback/link to available resources**: Facilitators provide feedback to mental health providers regarding implementation and action plan progress. Facilitators refer mental health providers to existing resources, including the Technical Assistance available through Standard REP, existing documents regarding the program intervention, facility-level, regional, or national leadership.	
March 1, 2013–August 2013	**Sites randomized to Standard REP who continue non-response receive 6 months of Enhanced REP Facilitation, remaining sites continued with Standard REP**	

After an additional 6 months, sites in VISNs initially randomized to receive Standard REP and who were still non-responsive (<80% documentation of patients’ current status) received Enhanced REP Facilitation from March 1, 2013 through August 31, 2013, described below and in Figure 1. Sites in VISNs that were initially randomized to receive Standard REP and *met* the implementation benchmark continued to receive Standard REP from March 1, 2013 through August 31, 2013. Sites in VISNs randomized to receive Enhanced REP in the first 6 months received Standard REP in the latter 6 months regardless of responsiveness. Note that while the two interventions being compared are adaptive (Enhanced versus Standard REP), the experimental study design is not an adaptive design, nor a cross-over design.

### Adaptive implementation strategies

Adaptive implementation strategy 1: *Standard REP*, described previously [37,42], consists of dissemination of a Re-Engage package describing the program’s core components, training the mental health providers implementing Re-Engage, and brief technical assistance offered to mental health providers that was designed to answer questions about specific Re-Engage components (Table 2). Training and technical assistance was provided through regularly scheduled national phone calls between March 2012 and August 2012 by staff members from the investigator team.

Adaptive implementation strategy 2: *Enhanced REP* included the addition of Facilitation, which has been defined as “a process of interactive problem-solving and support that occurs in the context of a recognized need for improvement and a supportive interpersonal relationship” [45]. Since Re-Engage was to be implemented nationally and is a relatively straightforward treatment delivered primarily by one provider at each facility, an External Facilitation role was based out of a central coordinating center [46]. Facilitation was delivered via telephone contacts for 6 months by three doctoral-level staff members with backgrounds in clinical psychology or counseling and were trained in the VA’s Blended Facilitation Model [47]. Individual semi-structured calls were made to the mental health providers at each facility, as well as to leaders in the facility’s regional network. Calls lasted on average about 30 min and occurred approximately one to three times per month for each facility.

The primary goal of Facilitation was to support the implementation of Re-Engage and included the following specific components [48] described in Table 2. Facilitators first gathered information using various sources to better understand the context of individual sites and reviewed internal reports generated from the web-based registry on Re-Engage implementation. Facilitators then scheduled individual calls with site providers to coach them on the implementation of Re-Engage. In addition, Facilitators contacted and built relationships with mental health leaders from the facility’s regional VA network to promote the Re-Engage program, support implementation, identify potential barriers, and if the site mental health provider desired, help resolve barriers to program adoption. Finally, Facilitators worked with the site providers on plans to overcome barriers to Re-Engage implementation and continued to provide feedback.

### Outcomes

Twelve-month outcomes assessed at the patient level included Re-Engage implementation and utilization of VA health-care services. Re-Engage program implementation was assessed using the three measures ascertained from the web-based registry: updated documentation of patients’ current status, attempted contact of the patient, and completed contact. Updated documentation was captured by determining whether the providers added in current information on the veteran’s clinical and social disposition based on the most recent VA medical record entries as well as available information regarding the veteran’s location and contact information via the internet. Attempted contacts were also recorded by the providers in the registry if they tried calling the veteran, calling his or her next of kin if their contact information was available, and if needed, sending a letter to the last known address of the veteran. Completed contacts were defined as documentation in the registry that the veteran was successfully reached to determine preference and need for services. All three measures involve clinical decision-making because the providers had to review clinical records in detail to understand the current disposition of the veteran and their relative need for care based on current symptoms and health status. As part of these contacts, providers were expected to make an immediate assessment over the phone pertaining to safety, acute symptomatology, or other clinical issue experienced by the veterans. These processes are akin to primary care clinicians reviewing medical records prior to a patient’s visit, and using available clinical data on diagnoses and past treatment history, determine who needs to be seen sooner rather than later.

Utilization outcomes post-randomization included any inpatient or outpatient visit within 12 months of initial randomization to Enhanced or Standard REP. Patient utilization data were ascertained from VA inpatient and outpatient administrative data available at the national level [49] using previously established methods [50,51]. These methods allowed the research team to summarize the total number of outpatient mental health and general medical visits (based on 300 or 500 series VA stop codes, respectively) and any inpatient visit in the VA system. All six patient-level outcomes—updated status, attempted contact, completed contact, any outpatient, any inpatient, and any outpatient or inpatient visit—are dichotomous, i.e., “yes” versus “no”.

### Analysis of primary and secondary aims

Descriptive statistics and bivariate analyses were conducted to compare sites starting on Enhanced versus sites starting on Standard REP based on patient- and site-level pre-randomization measures. For each outcome, we compared Enhanced REP versus Standard REP overall and in 6-month intervals (to account for immediate versus delayed effect of Enhanced REP) using a three-level mixed effects logistic regression model that accounted for the clustering of patients within sites within VISNs [50]. All regressions included an intercept, dichotomous indicator for Enhanced REP versus Standard REP, and independent random intercepts for site and VISN (assumed to follow a normal distribution). We further adjusted for the following *a priori* selected list of patient-level pre-randomization covariates: patient age, gender, race, VA service connection, married, homelessness, schizophrenia or related disorders diagnosis, number of medical comorbidity diagnoses, and whether last encounter was inpatient, as well as site-level factors including facility size (measured as number of unique patients in FY12), whether the site was an outpatient clinic or VA medical center (with at least one inpatient unit), the total number of patients with serious mental illness identified on the list to have dropped out of care (who were last seen at that site). The rationale for adjusting for these covariates is based on prior data suggesting these factors are associated with responsiveness to the Re-Engage program based on the original study [37]. For each outcome, we report both the crude and covariate-adjusted odds ratios of a “yes” value.

Power calculations have been described previously [37] and were originally based on a two-sample comparison of facilities within VISNs randomized to Enhanced versus Standard REP. Assuming a between-VISN variation (ICC = 0.177), and a two-sided, two-sample *t* test based on a type-I error rate of 5%, we had 80% power to detect an effect size of 0.72 (Cohen’s D). However, the current study is a secondary analysis designed to identify potential benefits of Enhanced REP based on multilevel models. Notably, sample size calculations were not performed for patient-level outcomes.

## Results

By August 2012, 89 sites were identified as having <80% uptake (updated documentation of patients’ current status), and these sites were randomized to Enhanced or Standard REP. The average percentage of updated documentation across the initially non-responsive sites (*N* = 89) was 24.0% (SD = 27.4%), and among the sites initially randomized to Standard REP (*N* = 49) was 22.6%; (SD: 28.1%) and for sites randomized to continuation of Standard REP was 25.1% (SD = 27.0%).

Among sites randomized to Enhanced versus Standard REP, there were no significant differences in patient-level characteristics with the exception that patients from Enhanced REP sites were less likely Black and less likely to have a diagnosis of schizophrenia or related disorder (Table 1).

**Figure 2:**
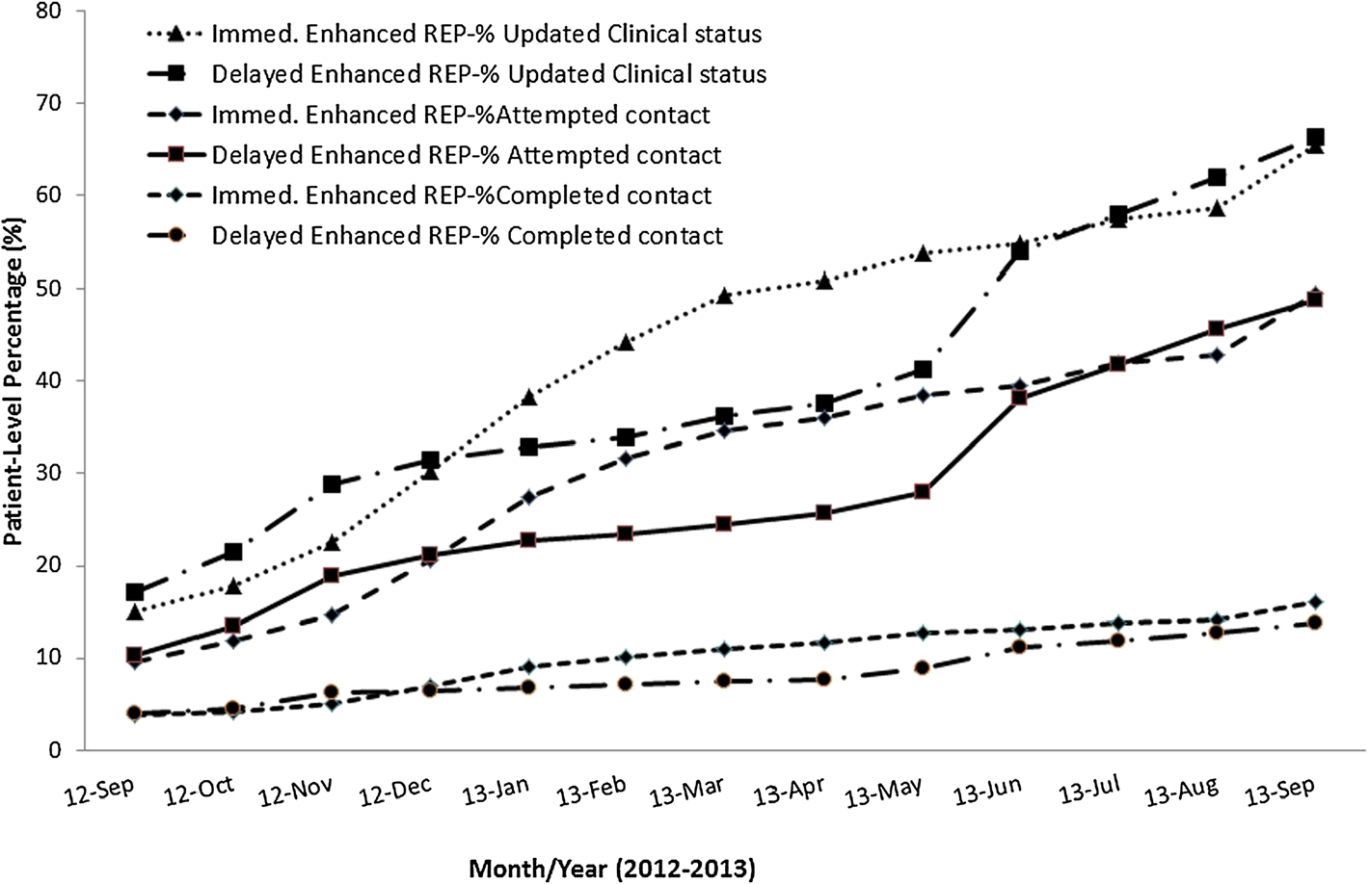
**Re-Engage program uptake over the 12-month period comparing adaptive implementation strategies (immediate versus delayed Enhanced REP).**

Multivariable results after adjusting for additional patient and site factors showed that there was a statistically significant difference in Re-Engage program uptake when Enhanced REP was applied immediately (Table 3). In particular, patients from non-responsive sites receiving Enhanced REP in the first 6 months of randomization compared to Standard REP sites were more likely to have a completed contact (adjusted OR = 2.13; 95% CI: 1.09–4.19, *P* = 0.02). Delayed Enhanced REP was also significantly associated with program uptake, where patients from sites that were no longer receiving REP after 6 months were less likely to have their clinical status updated compared to sites receiving Enhanced REP over the 6–12-month study period (Table 3). Enhanced REP was not associated with statistically significant probability of Re-Engage program uptake over the 12-month period, although the overall trend was increased program uptake (Figure 2).

**Table 3:** **Patient-level response to implementation of Re-Engage program comparing non-responsive sites randomized to one of two implementation strategies (Enhanced or Standard Replicating Effective Programs (REP))**

	**Implementation strategy number 1: patients from non-responsive sites randomized to first receive 6 months of Enhanced REP (immediate) (** ***N*** **= 1,543)**	**Implementation strategy number 2: patients from non-responsive sites randomized to first receive 6 months of Standard REP (delayed) (** ***N*** **= 1,532)**	**Unadjusted OR (95% CI)**	**Adjusted OR** ^**a**^ **(95% CI)**
Cumulative outcomes assessed over the 12-month period (August 31, 2012–August 31, 2013)				
Updated status	848 (54.96%)	613 (40.01%)	1.18 (0.43, 3.28)	1.29 (0.43, 3.90)
Attempted contact	694 (44.98%)	491 (32.05%)	1.06 (0.44, 2.59)	1.13 (0.44, 2.93)
Completed contact	198 (12.83%)	142 (9.27%)	1.26 (0.72, 2.18)	1.31 (0.70, 2.43)
Health-care use				
Any outpatient use	358 (23.20%)	382 (24.93%)	0.90 (0.70, 1.16)	0.94 (0.72, 1.22)
Any inpatient use	56 (3.63%)	58 (3.79%)	0.97 (0.62, 1.54)	1.00 (0.66, 1.51)
Any inpatient or outpatient use	361 (23.40%)	383 (25.00%)	0.91 (0.71, 1.17)	0.94 (0.72, 1.23)
Outcomes in first 6 months of implementation intervention (August 31, 2012–February 28, 2013)				
Updated status	605 (39.21%)	262 (17.10%)	2.85 (0.99, 8.25)	2.81 (0.93, 8.54)
Attempted contact	479 (31.04%)	207 (13.51%)	2.15 (0.81, 5.71)	2.21 (0.80, 6.05)
Completed contact	121 (7.84%)	57 (3.72%)	1.94 (1.01, 3.74)*	2.13 (1.09, 4.19)*
Health-care use				
Any outpatient use: August 31 2012–February 28 2013	234 (15.17%)	256 (16.71%)	0.89 (0.69, 1.16)	0.95 (0.73, 1.24)
Any inpatient use	40 (2.59%)	43 (2.81)	0.89 (0.54, 1.46)	0.89 (0.53, 1.49)
Any inpatient or outpatient use	237 (15.36%)	257 (16.78%)	0.91 (0.70, 1.17)	0.96 (0.74, 1.25)
Outcomes in second 6 months of implementation intervention (March 1, 2013–August 31, 2013)				
Updated status	243 (15.75%)	351 (22.91%)	0.30 (0.11, 0.84)*	0.33 (0.12, 0.91)*
Attempted contact	215 (13.93%)	284 (18.54%)	0.39 (0.15, 1.08)	0.43 (0.16, 1.19)
Completed contact	77 (4.99%)	85 (5.55%)	0.58 (0.26, 1.30)	0.61 (0.25, 1.49)
Health-care use				
Any outpatient use: March 1 2013–August 31 2013	276 (17.89%)	290 (18.93%)	0.96 (0.72, 1.28)	0.99 (0.74, 1.34)
Any inpatient use	28 (1.81%)	30 (1.96%)	0.92 (0.51, 1.65)	0.99 (0.56, 1.72)
Any inpatient or outpatient use	278 (18.02%)	291 (18.99%)	0.96 (0.72, 1.28)	0.99 (0.74, 1.33)

^a^Adjusted odds ratios (OR) were obtained from multilevel hierarchical logistic regression models after adjusting for the effects of the following covariates: patient characteristics (age, gender, race, marital status, VA service connection, homelessness, schizophrenia diagnosis, whether last encounter was inpatient, number of medical comorbidity diagnoses) and site characteristics (outpatient clinic status, facility size, and total number of patients on the original list). These models considered patients clustered within sites, and sites are nested within VISNs.

*P value < 0.05.

Overall, Enhanced REP was not associated with cumulative differences in utilization after adjustment over the 12-month period.

## Discussion

This paper describes to our knowledge one of the first trials comparing two adaptive implementation interventions. The study tested the effectiveness of immediate versus delayed deployment of Facilitation as an adjunct to Standard REP on the implementation of a national VA program, Re-Engage, which is designed to assist patients with serious mental illness who have been lost to care return to VA services. We found that among sites initially not responding to REP in 6 months, those that were randomized at the regional network level to receive Enhanced REP immediately (added Facilitation) or 6 months later were more likely to adopt Re-Engage. However, the cumulative effects of Enhanced REP over the 12-month period were not statistically significant.

To date, this was one of the first studies to employ an adaptive implementation intervention. In doing so, we were able to use a study design that took advantage of the national rollout of a clinical mandate. Although national mandates alone are limited in their ability to implement programs due to their top-down nature [52-54] conducting the present trial in the context of a clinical mandate provided additional support for the implementation of Re-Engage across sites [42]. Moreover, Re-Engage is one of the first brief interventions to be implemented for people with serious mental illness at a national level, using national data and web-based reporting registries to document program uptake and patient status. Ultimately, Standard REP and Enhanced REP were well-matched to the implementation of Re-Engage because the components were designed to be employed across multiple sites via internet and phone, which enhances the potential for scalability. This approach also allowed for the rollout of implementation strategies on a national level, thus potentially saving travel and personnel costs [55-58].

Nonetheless, we found that Enhanced compared to REP did not result in an increased proportion of patients returning to care or increased utilization of services among those who had dropped out of care. There are several reasons for these findings. First, not all patients were able to be contacted, and even fewer had a completed contact that would have routed them to services. In contrast, over two thirds of patients in the original study [37] were successfully contacted and re-engaged into services. The length of time patients were lost to care was longer than in the present study (maximum of 4 years and 5 months) than the length of time lost to care in the original assessment (maximum of 3 years). In addition, the time period for measuring return to care was longer in the original study (2 years), while this study only examined 1-year utilization outcomes. Perhaps patients who had dropped out of care longer ago were less likely to have been found by providers. In addition, historical trends in preference for VA care might have changed since the initiation of the original effectiveness study (2007 versus 2012). Perhaps patients who were reached may not have desired care at the time of contact or had access to care external to the VA.

Another reason for the null findings regarding utilization may have been due to the randomization at the regional network level as opposed to site level. The study was designed with the assumption that Facilitation involved contacts with regional network leadership levels, which would have made randomization at the site level vulnerable to potential contamination. Nonetheless, a key component of Enhanced REP was the ability to coach individual providers in a relatively efficient manner (via phone calls) rather than involving multiple levels of leadership and frontline staff to implement quality improvement initiatives, which can be expensive, time-consuming, and often require buy-in from multiple stakeholders. The central underpinning of Facilitation is the ability to empower the clinicians ultimately responsible for implementing a new initiative. Hence, while Facilitation included contacts with regional leadership in order to promote Re-Engage, in reality, they were primarily focused on addressing concerns brought up by the individual providers at each site, especially regarding lack of time to contact patients and difficulty in locating them [58]. Hence, the three-level cluster analysis (VISN, site, patient) represented a more conservative estimate of effects given more limited power with 20 networks. Moreover, while it is possible that effect of Facilitation might have been because of increased administrative attention through leadership contacts, much of this attention may have occurred prior to when Facilitation was implemented because the national directive was mandated and rolled out to VA regional mental health leaders in March 2012, approximately 6 months prior to initiation of added Facilitation (August 2012).

There are limitations to this study that warrant consideration. We were unable to assess utilization of services outside the VA, as VA administrative data do not capture other types of services such as state-run, not-for-profit, or private community-based clinics or mental health programs. Although it would have been scientifically informative to have continued Enhanced REP for greater than 6 months, the rapid implementation of Re-Engage was a high priority of VA leaders. Hence, the study was designed so that all sites could have access to the potential benefits of Enhanced REP in a relatively short period of time. Transportation costs and the national cohort of VA sites precluded our ability to provide in-person REP components (e.g., training) or Enhanced REP Facilitation, which could potentially have increased the potential impact of the implementation strategies [33,59]. The national rollout of the Re-Engage clinical program via a VA Directive precluded our ability to measure provider behavior or other clinical processes of care (e.g., qualitative assessments or taping contacts with veterans). Finally, the Enhanced REP Facilitation strategy was mainly focused the adoption of a relatively straightforward clinical protocol, whereas a more comprehensive improvement strategy (e.g., systems engineering) might be warranted for more complex health services interventions involving the adoption of care processes across multiple interdisciplinary teams of providers [60,61].

## Conclusions

Overall, Enhanced REP was associated with greater uptake of an outreach program designed for patients with serious mental illness among sites initially not responding to a standard implementation strategy. Enhanced REP was most effective when applied immediately among sites not responding to a standard implementation strategy. Further research is needed to determine whether national implementation of Facilitation results in tangible changes in patient-level outcomes including utilization and quality of care. Additional analyses regarding relative costs of enhanced implementation strategies are also warranted. Finally, adaptive implementation intervention designs may hold promise in the more efficient allocation of implementation support notably for sites that are not responsive to less intensive implementation approaches to facilitate the uptake of evidence-based practices.

## Abbreviations

REP: Replicating Effective Programs framework
PARiHS: Promoting Action on Research Implementation in Health Services framework
SMI: Serious mental illness
VA: Veterans Health Administration
ICD-9-CM: International Classification of Diseases
Ninth Revision: Clinical Modification
VISN: VA integrated service network
